# Seafood consumption and persistent organic pollutants as triggers of autoimmunity among Gullah African Americans

**DOI:** 10.1186/ar3953

**Published:** 2012-09-27

**Authors:** DL Kamen, MM Peden-Adams, JE Vena, GS Gilkeson, TC Hulsey, L Moultrie, BE Stevens

**Affiliations:** 1 Medical University of South Carolina, Charleston, SC, USA; 2 Hollings Marine Lab, Charleston, SC, USA; 3 College of Public Health, University of Georgia, Augusta, GA, USA; 4 Sea Island Families Citizen Advisory Committee, Charleston, SC, USA; 5 Eastern Virginia University, Norfolk, VA, USA

## Background

Local seafood is a dietary staple among the African American Gullah population of South Carolina. High levels of persistent organic pollutants (POPs) have been found in local bottlenose dolphins, sentinel species for human health and consumers of many of the same fish as the Gullah. Links have been established between these bioaccumulating, ubiquitous compounds and deleterious health effects in humans. The objective was to determine whether levels of POPs, specifically perfluorinated compounds (PFCs), correlate with fish intake and markers of immune dysfunction in genetically at-risk individuals.

## Methods

At the onset of the Persistent Organic Pollutants in AutoImmunity (POPAI) study, one-on-one interviews were conducted with Gullah community members to validate a comprehensive environmental exposure questionnaire. The validated questionnaire, including a seafood intake survey, was then administered prospectively to patients with lupus, first-degree relatives of lupus patients, and unrelated nonlupus controls participating in the SLE in Gullah Health (SLEIGH) study. PFC levels (PFOS, PFOA and PFNA), antinuclear antibody titers and other autoantibodies were measured in the serum of participants drawn at the time of their study visit.

## Results

Seafood intake questionnaires received from 103 Gullah participants enrolled in the SLEIGH study found 57% consumed locally caught seafood at least once a month and 40% consumed species known to contain high levels of POPs in the Charleston Harbor area. Preliminary results from 33 Gullah controls show that all have measurable serum levels of PFCs (specifically PFOS, PFOA and PFNA) from baseline and follow-up visits 7.3 ± 1.4 years apart, with annual servings of seafood directly correlating with serum PFOS and PFNA (p=0.02 and 0.03). ANA positive controls (48% at baseline) had higher mean levels compared to ANA negative controls for PFOS (75.1 vs 48.2 ng/ml, p=0.06), PFOA (7.0 vs 5.8, p=NS) and PFNA (3.2 vs 2.1, p=0.04).

## Conclusion

These ongoing studies address concerns of the Sea Island Gullah community regarding the potential immune health effects of the bioaccumulating pollutants found in local dietary staples such as fish.

